# Upper extremity phlegmasia cerulea dolens complicating supra vena cava embolism in a cervical cancer patient: case report

**DOI:** 10.3389/fcvm.2024.1351358

**Published:** 2024-02-06

**Authors:** Jinting Ge, Chengxin Weng, Jichun Zhao, Ding Yuan, Tiehao Wang

**Affiliations:** Division of Vascular Surgery, Department of General Surgery, West China Hospital, Sichuan University, Chengdu, China

**Keywords:** phlegmasia cerulea dolens, venous gangrene, upper extremity, supra vena cava embolism, pulmonary embolism, cervical cancer

## Abstract

Phlegmasia cerulea dolens (PCD) is a rare yet severe complication of deep vein thrombosis (DVT), characterized by a high amputation rate and mortality. Early diagnosis and treatment are crucial in managing this condition. PCD predominantly affects the lower extremities rather than the upper extremities. We herein present a rare upper extremity PCD case accompanied with supra vena cava and pulmonary embolism in a cervical cancer patient, who presented to our institution with severe pain, edema and irreversible venous gangrene of right upper limb with no response to anticoagulation therapy. Emergency fasciotomy and amputation were performed due to the progressed venous gangrene, however, the patient developed severe infection and coagulation disorders, gastrointestinal bleeding and disseminated intravascular coagulation after the surgery. Despite medical interventions, her family chose to withdraw treatment and the patient died in ICU at the fourth day following emergency surgery.

## 1 Introduction

Phlegmasia cerulea dolens (PCD) is rare and the most severe complication resulting from deep vein thrombosis (DVT) with high amputation rate up to 25%–50% and mortality of 30%, which could cause severe edema, intractable pain and irreversible venous gangrene (1–3). Therefore, early diagnosis and intervention of PCD is vital as PCD progresses rapidly and may result in ischemic events and the need for amputation (3). Currently, most reported PCD cases were described in the lower extremities, while only 2%–5% of PCD occurred in the upper extremities (1, 3–5). We herein present a case of upper extremity PCD involving supra vena cava and pulmonary embolism, who presented with massive edema, severe pain and venous gangrene of right hand and forearm.

## 2 Case presentation

**Figure 1:**
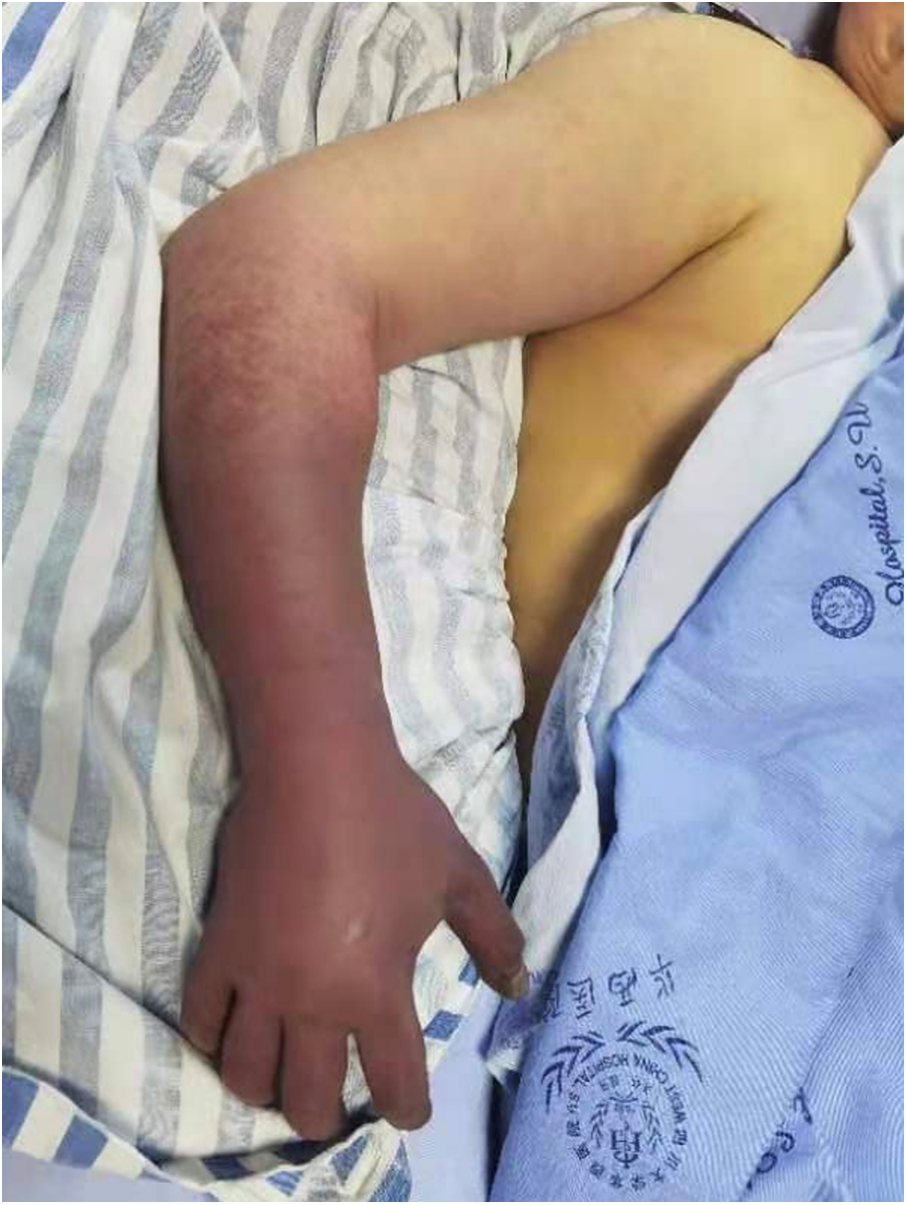
Appearance of right upper extremity on admission.

A 40-year-old female presented to our hospital with a chief complain of severe pain and edema in right forearm and hand for 5 days. She had been suffering recurrent mild edema and pain in bilateral lower extremities for the last month. Local hospital found no signs of heart or kidney diseases. The patient then developed intractable pain and massive swelling in right upper extremity and further progressed to coldness, cyanosis, paresthesia and paralysis 2 days before admission (Figure 1). Additional symptoms included cough and dyspnea. Past medical history showed that the patient was diagnosed with stage IIIB cervical cancer 6 months ago and received peripherally inserted central catheter (PICC) implantation as well as multiple rounds of chemo- and radiotherapy. However, her PICC was removed 2 months ago without any reported thrombosis events noted. The patient also had no hypertension, hyperlipemia, atherosclerosis, atrial fibrillation or coronary artery diseases.

**Figure 2:**
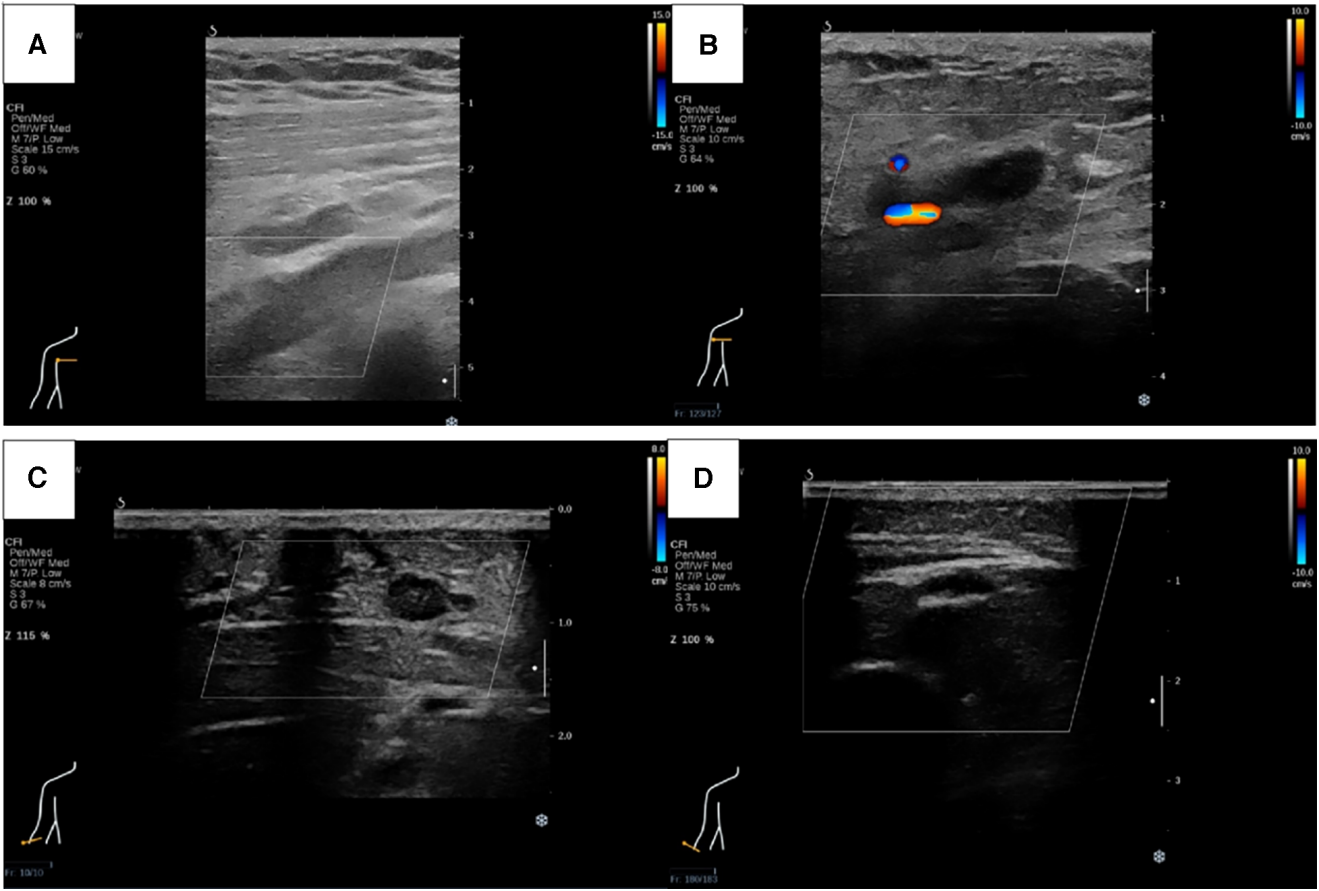
Ultrasound revealed thrombosis of right subclavian vein (**A**), axillary vein (**B**) and cephalic vein (**C**), as well as right radial, ulnar and part of branchial artery (**D**).

**Figure 3:**
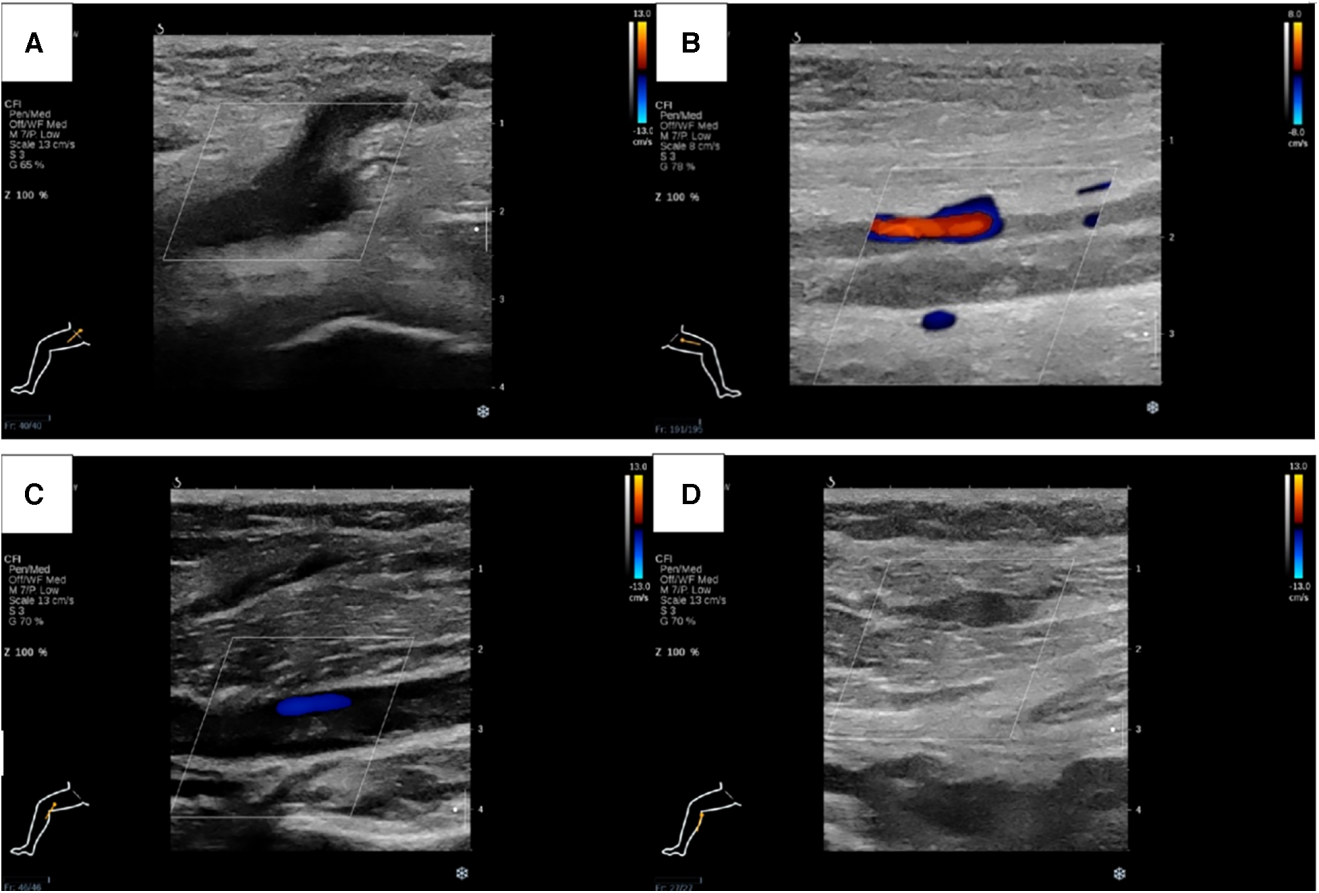
Lower extremity ultrasound demonstrated occlusive embolism of right common femoral vein (**A**), bilateral superficial femoral vein (**B**), right popliteal vein (**C**) and calf muscular vein (**D**).

**Figure 4:**
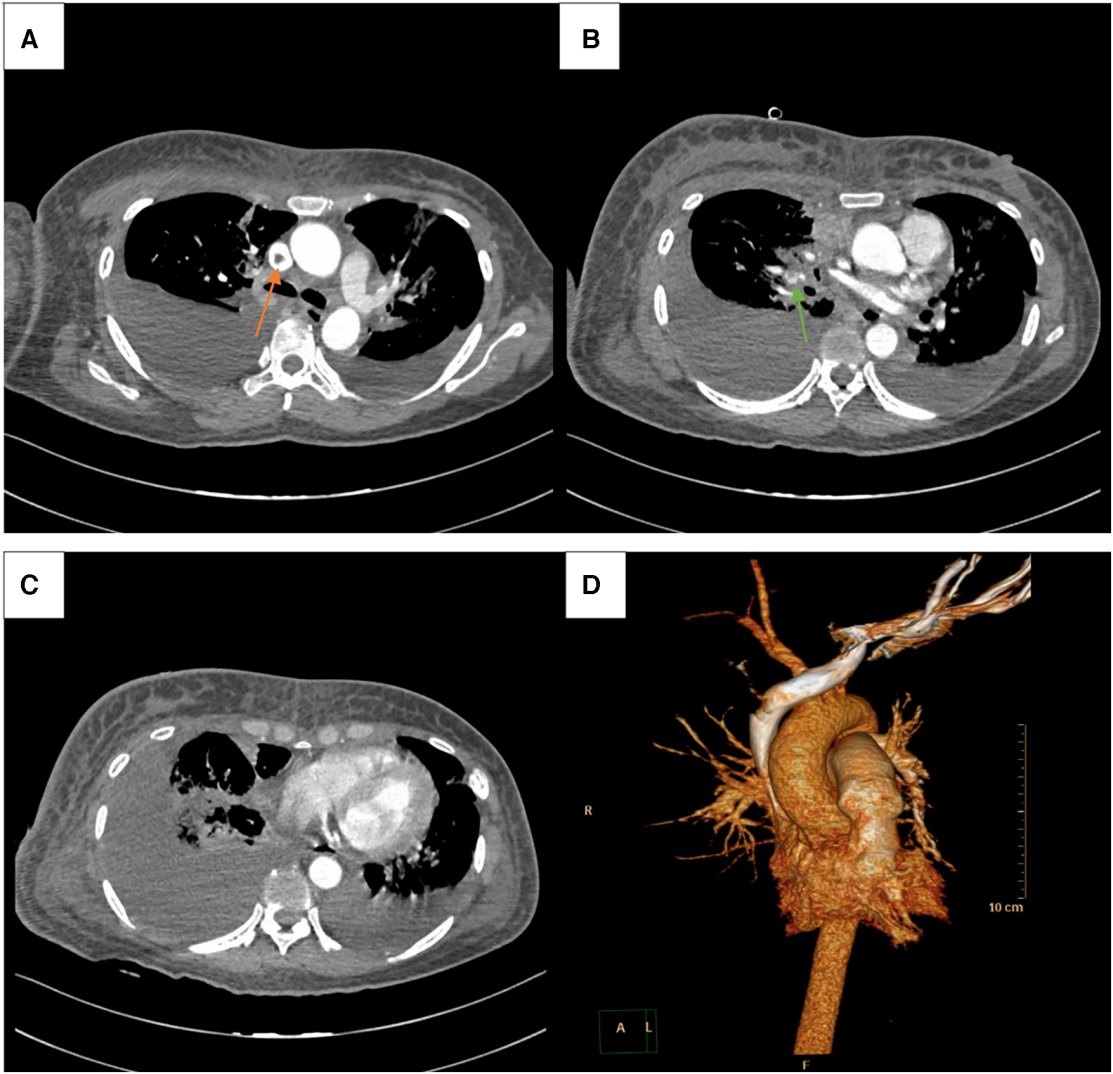
CTPA showed superior vena cava and pulmonary embolism (orange and green arrow, respectively) (**A, B**), along side with pleural effusion in bilateral chest, especially in right side (**C**). 3D reconstruction image of CTPA (**D**).

Physical examination (PE) revealed pulselessness of right radial and ulnar artery and high skin tension of right upper extremity. The patient exhibited complete loss of sensation in her right forearm and hand, along with an inability to move the right joint wrist, metacarpophalangeal wrist and finger joint. Duplex ultrasound for upper extremity vessels showed extensive and complete thrombosis of right subclavian vein, axillary vein and distal veins (Figures 2A–C). There were no arterial signals over the right radial, ulnar and part of branchial artery on ultrasound (Figure 2D). Meanwhile, lower extremity ultrasound demonstrated occlusive embolism of right common femoral vein, bilateral superficial femoral vein, right popliteal vein and calf muscular vein (Figures 3A–D). Computed tomography pulmonary angiography (CTPA) further revealed superior vena cava and pulmonary embolism (PE), alongside with pleural effusion in bilateral chest, especially in right side (Figures 4A–D). Initial lab tests revealed hemoglobin (HGB) of 59 g/L, white blood cell (WBC) of 19.07 × 10^9^/L, platelet (PLT) of 68 × 10^9^/L, creatinine of 104 umol/L, creatine kinase (CK) of 14,433 IU/L, blood potassium of 4.39 mmol/L, blood lactic acid (LAC) of 4.18 mmol/L, prothrombin time (PT) of 15.5 s, international normalized ratio (INR) of 1.40, activated partial thromboplastin time (APTT) of 35.8 s, fibrinogen (FIB) of 0.94 g/L, D-dimer of 23.78 mg/L, brain natriuretic peptide (BNP) of 458 ng/L and myoglobin of >3,000 ng/ml, protein C and protein S activity were normal. Therefore, diagnosis of right upper extremity PCD, supra vena cava and pulmonary embolism was proposed. According to the body weight of our patient (59 kg), anticoagulation (enoxaparin, 6,000 IU, q12h), antibiotics (tazocin, 4,500 mg, q8h) were prescribed and blood transfusion was arranged for the patient immediately. Chest tube drainage was also performed to alleviate cough and dyspnea. However, the right upper extremity of the patient became more swelling and painful and developed tension vesicles during examination. The sensation and ability to move of her hand and forearm did not improve after anticoagulation treatment, as well as respiratory symptoms. Thus, emergency fasciotomy was performed due to progressed venous gangrene, despite peri-operative risk was high due to low HGB, PLT and FIB counts, and the chance of limb salvage was relatively low because physical examination results and high WBC, CK and myoglobin counts suggested high extremity necrosis possibility. Extensive venous and arterial embolism were observed alongside muscle necrosis with no response to electrical stimulation, henceforth necessitating inevitable right forearm amputation. Rheolytic thrombectomy or catheter-directed thrombolysis (CDT) were not utilized because we believe the venous gangrene and limb necrosis were irreversible.

After the emergency fasciotomy and amputation, the patient was transferred to intensive care unit (ICU) for further treatment. Post-operative lab tests demonstrated HGB of 75 g/L, WBC of 24.59 × 10^9^/L, PLT of 60 × 10^9^/L, creatinine of 172 umol/L, CK of 21,049 IU/L, blood potassium of 5.51 mmol/L, LAC of 8.53 mmol/L, PT of 59.3 s, INR of 4.88, APTT of 92.7 s, FIB of 2.49 g/L, D-dimer of 34.29 mg/L, BNP of 773 ng/L and myoglobin of >3,000 ng/ml, which suggested that the patient developed severe infection and coagulation disorders in several hours after fasciotomy and amputation. In addition, the patient suffered from gastrointestinal (GI) bleeding at the following day and received emergency endoscopic hemostasis. Therefore, we had no choice but to suspend her anticoagulation therapy due to active GI bleeding and severe coagulation disorders. The patient then developed disseminated intravascular coagulation (DIC) with increased INR level of 8.6 and major bleeding in the next few days, her trusted proxy chose to withdraw treatment and the patient died in ICU at the fourth day after emergency procedure.

## 3 Discussion

DVT can result in partial or total outflow obstruction of venous system, leading to severe consequences. PCD represents the most extreme manifestation of DVT, which could lead to severe edema, pain, venous hypertension, intestinal pressure elevation and compartment syndrome in extremity and ultimately progress to arterial thrombosis and venous gangrene (1–3, 6). Current medical literature regarding PCD were mostly case reports and there is no literature that oversaw the exact incidence of PCD due to its rarity and lack of cohort study (3, 7). In addition, majority PCD cases involved lower extremity, while upper extremity PCD is extremely rare and account for less than 5% of all PCD cases (4,5). Malignancy, hypercoagulable state, infection, venous stasis, heart diseases and central venous catheter implantation had been identified as common risk factors of DVT and PCD in previous studies, moreover, heparin-induced thrombocytopenia and pacemaker were also reported to induce PCD and venous gangrene (3, 7–11). The association between venous thromboembolism (VTE) and malignancy has been well studied and described before. It is reported that the estimated incidence of VTE in cancer patients is 5 times higher than general population, moreover, cancer-related-thrombosis patients have poorer prognosis than normal VTE patients (12–14). As for our case, we believe that malignancy, hypercoagulable state and PICC implantation history were main causes of PCD and venous gangrene. It is worth noted that the patient presented with recurrent lower extremity edema and pain at 1 month before admission to our institution, later ultrasound confirmed the existence of lower extremity DVT. Unfortunately, the patient did not seek for medical advice or receive anticoagulation therapy, which may result in the progress of coagulation disorders and VTE and finally lead to PCD.

Although the overall case numbers were relatively low, the prognosis of PCD patients was poor with high morbidity and mortality (3, 7). Several risk factors had been found to be related to worse outcomes in PCD patients. A systematic review conducted by Greenberg et al. analysed the outcome of 37 upper extremity PCD cases and revealed that concomitant presence of lower extremity PCD was associated with higher mortality, moreover, bivariate analysis also found that bilateral upper extremity disease suggested a worse outcome, although no significant difference was observed (3). In addition, Chinsakchai et al. pointed out that malignancy was the most common risk factor of PCD and 55% of PCD patients who had underlying malignancy died eventually, while PE was reported as another risk factor and resulted in 50% mortality in PCD patients (7). At the same time, prior studies indicated that higher grade of PCD severity may be important predisposing factor of poorer outcome, patients with venous gangrene, also known as Grade III PCD, had higher associated morbidity and mortality rates than noncomplicated PCD (Grade I) or impending venous gangrene (Grade II) patients (7, 15,16). Our case had numerous risk factors associated with poor outcome in PCD, including medical history of malignancy, lower extremity DVT and PE, coagulopathy and venous gangrene. It is also worth noted that our patient was complicated with supra vena cava thrombosis, which was not reported in previous upper extremity PCD cases or cohort. Meanwhile, only few cases reported simultaneous upper extremity arterial thrombosis, in our case, right radial, ulnar and part of branchial artery thrombosis occurred simultaneously with right upper limb PCD (1, 3, 17). The authors believed that these complications aggravated the clinical manifestations of our patient and lead to worse outcome.

Currently, there is no clinical guideline for upper extremity PCD, however, early identification and treatment for PCD patients is vital because PCD may progress to venous gangrene rapidly and the therapeutic effect would gradually diminish as the diseases progress (7). Anticoagulation therapy and extremity elevation should be the cornerstone of treatment once the patient is diagnosed with PCD (3). However, more specific treatment may be required for PCD patients, including systematic thrombolysis, CDT and open venous thrombectomy. Previous studies have demonstrated that CDT is associated with a decreased incidence of post-thrombotic syndrome (PTS) and venous obstruction compared to systematic thrombolysis in patients with acute iliofemoral deep vein thrombosis (18). On the other hand, open venous thrombectomy has been shown to be associated with a lower incidence of PTS and venous reflux than systematic thrombolysis (18). However, there is currently insufficient data available to compare the outcomes of CDT and surgical thrombectomy. Furthermore, additional research and data are needed to determine the clinical outcomes of these treatment options in upper extremity DVT or PCD. Saydam et al. investigated the outcome of rheolytic thrombectomy in PCD patients, which was found to be less invasive, safe and effective strategy for early stage PCD (15). However, several case reports indicated that neither thrombolysis or thrombectomy may increase the chance of limb salvage in high grade PCD patients, especially venous gangrene (1, 4, 17). Early fasciotomy should be performed to reduce neurological complications, limb necrosis and amputation in the settings of PCD complicated with compartment syndrome (3). In our case, the patient presented with Grade III PCD, sensation loss and limb ischaemia, while venous hypertension did not relieve after anticoagulation therapy. Therefore, emergency fasciotomy was conducted due to severe compartment syndrome and arterial thrombosis. Although CDT or mechanical thrombectomy was considered for our patient, unfortunately, the patient developed irreversible venous gangrene and extremity necrosis, which left no room for thrombolysis or thrombectomy but major amputation. The patient then suffered gastrointestinal bleeding and severe coagulation disorder in ICU, we chose to withhold her anticoagulation and this may predispose our patient to DIC and ultimately her death.

The main novelty of our case is the rarity of upper extremity PCD and venous gangrene because less than 5% of PCD cases affected upper extremity (1, 3–5). In addition, only three cases reported simultaneous arterial thrombosis in upper extremity PCD patients, while supra vena cava thrombosis was not reported in previous upper extremity PCD cases or cohort, the authors believe that these complications further increased the rarity, originality and complexity of the case report (1, 3, 17). However, our case also had limitations. Emergency thrombolysis or thrombectomy was not performed for this patient due to her delayed admission and progressed venous gangrene, which ultimately led to major amputation and death of our patient. It is demonstrated that timely and effective surgical treatment is vital for the prognosis of PCD patients through this case.

In conclusion, upper extremity PCD is a rarity with high limb loss rate and mortality, which requires early diagnosis and timely intervention. Persistent coagulation disorder, PICC implantation history and malignancy may be the main causes of PCD. Systematic thrombolysis, CDT, open venous thrombectomy, rheolytic thrombectomy and fasciotomy are main treatment options for PCD patients, although more studies are required to determine the standard care for PCD. Nevertheless, aggressive treatment should be conducted for all patients with potential risk of PCD, regardless of presentation of related symptoms (3).

## Data Availability

The original contributions presented in the study are included in the article/Supplementary Material, further inquiries can be directed to the corresponding author.
